# Placenta Prævia with Mal-presentation—Hysterotomy for Rigid Os Uteri

**Published:** 1886-07

**Authors:** J. W. Hamilton

**Affiliations:** Hornsby’s Bend, Texas


### PLACENTA PRAEVIA WITH MAL-PRESENTATION: HYSTEROTOMY FOR RIGID OS UTERI.

*By J. W. Hamilton, M. D., Hornsby's Bend, Texas.*

(For Daniel's Texas Medical Journal.)

DR. W. T. COLLINS' case of Vaginal Hysterotomy calls to mind a case which came under my treatment last year. At 9 o'clock P. M., September 16th, 1885, I was called to see Mrs. J. A. multipara, age 24 years; found her in labor. Was informed that she had been having slight hemorrhage at intervals for some five weeks, which hæmorrhage had been rather profuse for several days; was informed that regular labor pains had begun twelve hours previous to my being called in. Immediately suspected placenta prævia; made digital examination ; found os dilated about the size of a half dollar, thin and extremely rigid. Found partial placenta prævia. I will say the case was not at full term, but about seventh month of gestation. Patient did not seem to be suffering much from loss of blood; notwithstanding there had been continual hæmorrhage for several days, but the woman was having very severe labor pains almost constantly, which was very exhausting. Endeavored to push placenta to one side and bring down the head and by that means check the hæmorrhage. I succeeded in pushing placenta aside, but instead of the head, I found an arm and shoulder presentation to deal with, which caused some delay ; but I succeeded in returning arm and bringing head down which owing to limited dilation of os, required some time and a good deal of manipulation; but the head once being brought down, accomplisded the object in view—that of preventing *escape* of blood and *checking* hæmorrhage to some extent. I then administered chloral hydrate x x grs., hoping to overcome the extreme rigidity which still existed but after an hour had elapsed, another examination being made, I found no change in her condition whatever. I then annointed os well with belladonna ointment and repeated applications in fifteen minutes, but without desired effect. About 12 o'clock I was told by one of the attendants that the patient was "jerking all over." Upon examination I found her with convulsions : the muscles of trunk and extremities jerking violently, which continued for more than a minute, after which the patient did not seem to recognize any one ; nor in fact to be rational at all. I repeated chloral hyd. grs. xxx, after which I proceeded to nick the border of the os with scissors to the depth of about ¼ inch at three different places, making one anterior and two lateral incisions, leaving posterior portion of os unbroken, so that the "cleanings" and lochial discharges could pass off without coming in contact with the broken surface, thereby obviating the dangers of septic poisoning from that source. After this, I succeeded in delivering fœtus and placenta in a very short time. There being considerable loss of blood after delivery, I administered "Squibbs" Fl. Ext. Ergot ℨ. 1. Tr. Digitalis min. xx, and proceed to syringe out uterus with a strongly carbolized sol. of one part water, two parts strong vinegar as warm as patient could bear. Hemorrhage ceased; womb contracted well; patient resting well and apparently in good condition. Prescribed dose of Epsom salts to be taken the following day, also six grains quinine to be given three times a day for several days. Mixture ergot and digitalis to be taken should hemorrhage occur again. Patient did well until eighth day. She had a chill followed by fever, suppression of lochia, headache and considerable pain over the womb. Ordered carbolized vaginal injections twice daily and prescribed quinine x grs. every four hours; fever subsided and patient made a good recovery.

While "nicking of os" to overcome rigidity of that part in labor is recognized by many of our modern writers and taught in our best medical schools, it is surprising that it is not practiced more extensively than it is. It is an operation that does not require any great skill on the part of obstetrician, nor is it attended with any danger to the patient. Its advantages over other means of relieving rigidity of os cannot be disputed; it is better than chloroform because it is safer; it does not subject the patient to the dangers of hemorrhage which the former agent does. It is better than a sponge tent or a Barnes dilator because it is much quicker; it enables us to deliver without delay, and certainly where hemorrhage is an alarming symptom it is always advisable to deliver as soon as possible.

[We commend Dr. Hamilton's article and that of Dr. Collins in our last number, to certain physicians in the northern part of this State. We see an account of a case of "rigid os" reported in the MEDICAL WORLD recently in which five physicians were divided between embryotomy and Cæsarean section; but they agreed upon the former, and removed the child piece-meal—the mother died. These gentlemen in reporting the case ask for suggestions as to what was best to have been done under the circumstances. If labor was actively progressing and there was any *especial reason* for terminating it then and there, possibly the plan adopted by Drs. Hamilton and Collins referred to above, would have been attended with better result than the one employed—but we have seen similar cases come around all right if left alone a while; say—under a small tranquilizing potion, or a full dose of some opiate,—labor arrested—patient recuperated in strength and cheered up a little—work resumed next morning—with proper dilatation and easy delivery. We would have *waited*, we believe, in the case reported by Drs. Thompson and Moody, in the WORLD. Spontaneous evolution or version has not unfrequently occured, and the arm presentation was not a contra indication to waiting—certainly not to "nicking".—ED.]

